# Debriefing practices in interprofessional simulation with students: a sociomaterial perspective

**DOI:** 10.1186/s12909-016-0666-5

**Published:** 2016-05-17

**Authors:** Sofia Nyström, Johanna Dahlberg, Samuel Edelbring, Håkan Hult, Madeleine Abrandt Dahlgren

**Affiliations:** Department of Behavioural Sciences and Learning, Linköping University, Linköping, Sweden; Department of Clinical and Experimental Medicine, Linköping University, Linköping, Sweden; Department of Medical and Health Sciences, Linköping University, Linköping, Sweden; Department of Learning, Informatics, Management and Ethics, Karolinska Institutet, Stockholm, Sweden

**Keywords:** Simulation, Undergraduate health professions education, Multiprofessional, Professionalism, Medical education research methodology

## Abstract

**Background:**

The debriefing phase is an important feature of simulation activities for learning. This study applies a sociomaterial perspective on debriefing in interprofessional simulation with medical and nursing students. Sociomaterial perspectives are increasingly being used in order to understand professional practice and learning in new ways, conceptualising professional practice as being embodied, relational and situated in sociomaterial relations. The aim of the study is to explore how debriefing is carried out as a practice supporting students’ interprofessional learning.

**Methods:**

Eighteen debriefing sessions following interprofessional full-scale manikin-based simulation with nursing and medical students from two different universities were video-recorded and analysed collaboratively by a team of researchers, applying a structured scheme for constant comparative analysis.

**Results:**

The findings show how debriefing is intertwined with, and shaped by social and material relationships. Two patterns of enacting debriefing emerged. Debriefing as *algorithm* was enacted as a protocol-based, closed inquiry approach. Debriefing as laissez-faire was enacted as a loosely structured collegial conversation with an open inquiry approach.

**Conclusion:**

The findings indicate that neither an imposed structure of the debriefing, nor the lack of structure assured interprofessional collaboration to emerge as a salient topic for reflection, even though that was an explicit learning objective for the simulation.

## Background

This study explores debriefing as a means of learning interprofessional collaboration skills through medical full-scale simulation practice, in the context of undergraduate medical and nursing education. Seen in a global perspective, interprofessional collaboration skills have been pointed out in policy documents as necessary for health professionals in order to meet future health care demands. Interprofessional collaboration is seen as a means of increasing the efficiency and capacity of the health care workforce [1]. Consequently, there is also a global call for to incorporate interprofessional learning activities in professional health care education curricula [2]. Simulation is increasingly being used for the training of interprofessional collaboration skills in undergraduate medical and nursing programs. Some studies indicate that student interprofessional simulation improves role clarification and promotes problem-solving skills [3]. However, research reviews show that the impact of such training is still under-researched [4].

The model for simulation-based education is generally structured according to three phases. The first phase, the briefing, provides information on the technical equipment in use and the scenario that is about to be simulated. The second phase, the actual simulation, is where participants enact the scenario in practice as if it was an authentic clinical case. In the third phase, the debriefing, participants’ reflection are promoted. Participants’ emotional reactions, actions and interactions in the scenario are often brought up as topics. The debriefing phase is viewed as being critical for the participants’ learning in a simulation [5–9]. Fanning and Gaba [10] clarify the bridging function that debriefing serves between experiencing an event and learning from it. Debriefing can be seen as a social practice [11], where several dimensions and multiple purposes can be discerned.

The instructors’ ways of conducting the debriefing, and the probing questions to the participants to facilitate reflection are seen as crucial [12]. Dieckmann et al. [11] elaborate on the dynamics of instructor-participant interaction, suggesting that different instructor styles influence the process and outcomes of the debriefing. In a recent study, Husebø et al. [13] found that the instructors’ probing questions in the debriefing are often too descriptive to serve the learning process. There is a need, the authors argue, to develop a more analytic approach to probing questions in order to facilitate deeper reflection. Other aspects of debriefing, such as various debriefing techniques, the quality and degree of instructor training, and the effects of different debriefing venues and times, have also been pointed out as areas in need of more research [5].

Historically, debriefing in the military and aviation domains follows up on crisis situations in order to receive an accounting of a mission, and allows the participants to express and discuss emotions to mitigate their stress [10, 14]. Dreifuerst and Decker describe how debriefing has migrated to a variety of fields, and propose that debriefing in health care serves three purposes: “…*to receive an accounting, to mitigate emotional response, and to correct decisions and actions that were incorrectly applied in the simulation experience*” ([14], p. 106). The migration of simulation into the health professions has been a successful one if measured by traditional metrics [15]. However, there are reasons to further investigate simulation and debriefing as pedagogical practices. Taking a pedagogical perspective on the literature of simulation, Rooney et al. [16] discerned three focal themes of interest concerning 1) the fidelity of the simulation, 2) the linking of simulation to outcomes in practice, and 3) pedagogical underpinnings. The authors characterise the theoretical basis as being predominantly evaluative and protocol-driven. Reamer et al. [5] also emphasise the need for more critical research on the debriefing process by exploring: who is debriefing, what is the content and method, the timing (when), the environment (where) and the supporting theoretical framework (why) (p. S53). There is a need to further explore and expand the conceptualisation of simulation-based education in general in order to understand how to best support students’ learning for work [16].

Recent research and theories on work and learning have implied that individualist and cognitive perspectives are not sufficient for understanding learning in a professional practice. Stocker et al. identify a similar lack of theoretical understanding of simulation for the purpose of team training, proposing a synthesis of learning theories incorporating social and cultural aspects [17]. Other authors have more specifically suggested that practice-oriented perspectives on professional learning [18] are needed to address the increasing complexity of contemporary and future health care. Practice-oriented perspectives recognise the professional practice of health care as being embodied, relational and situated in sociomaterial relations. Practice perspectives are increasingly being used in order to understand professional practice and learning in new ways [18–21]. A study by Ahn et al. [22] showed that the three phases of simulation and their physical location in different rooms and specific social and material arrangements brought about different teaching practices and different possible learning outcomes for the participants. Using practice-oriented perspectives, the researcher can uncover complex courses of events and relations in practice that go beyond abstract outcome measures and experimental comparisons. Hence, the conceptualisation of debriefing in this paper is that of a practice which is relational to the social and material arrangements. The aim of the study is to explore debriefing as a practice intended to support students’ interprofessional learning.

### Theoretical framework

Taking a practice-oriented perspective means that simulation is viewed as an organised set of actions embedded in a practice. These organised sets of actions are interconnected through general understandings of the practice as a whole, but also guided by explicit and implicit rules and ethical considerations. An important difference to cognitive perspectives is that both human and non-human actors are taken into account in order to explain and understand how a nexus of actions emerges in a practice situation. The context is thereby not viewed as a passive container for the actions. Instead, materials and things are seen as dynamic and integrated with human actions in ways that act on human practice. An example is when automatic blinds regulate the daylight in a lecture room, making the teacher change from showing PowerPoint slides to writing on the whiteboard to compensate for the slides not being readable. A sociomaterial practice perspective takes into particular account the relationships between the theoretical concepts of *sayings*, *doings*, *material set-ups* and *relatings* of the situation. [23–25]. Kemmis & Grootenboer exemplify and develop these concepts as arrangements that build up what they call practice architectures. Human and non-human resources and *doings* can be viewed as *material-economic* arrangements of the practice. *Sayings,* professional language and conduct can be viewed as *cultural-discursive* arrangements, while the *relatings* between participants make up the *social-political* arrangement. These arrangements prefigure, i.e. they shape and are shaped by, the emerging practice [25].

It is important to emphasise that the focus in a sociomaterial perspective is not on singular things or technologies, but on *relationships* between human action and material arrangements, and how these relationships emerge in a practice [19, 26]. A focus on the social and material arrangements as relational can shed light on how and why certain activities become practically intelligible, i.e. more or less likely to happen in the unfolding practice [24].

## Method

This paper draws on empirical data gathered through video recordings and observational field notes on debriefing sessions held directly after simulation exercises. The contexts in this study are two sites (site 1 and site 2) of undergraduate education of health professionals, which are relevant for the study since they include simulation as part of their curricula. The sample comprised students participating in a full day of simulation exercises conducted at two university simulation centres in Sweden connected to teaching hospitals. As part of the activity, students were grouped into interprofessional teams that were kept together during the simulation day. At both sites, the interprofessional simulation was a compulsory part in the last semester before graduation. The stated aim of the simulation-based exercise was to provide opportunities for the students to engage in teamwork and interprofessional collaboration in a simulated clinical setting. All scenarios included in the simulation were variations on the themes of acute emergency or deteriorating condition of the patient. The students at both sites had previously learned about acute emergency care. The simulations as well as the debriefings were recorded in their naturalistic setting (i.e. not designed by the researchers). These settings varied in their layout as well as the time allocated.

The data is based on observations from 18 debriefing sessions from two different sites, comprising around five hours of video recordings. Altogether 106 students - 71 females and 35 males - participated in the debriefing, either as active participants (two medical students and two to four nursing student per scenario) or as observers (four to six students per scenario) in the simulation scenario. Sixty-six were nursing students and 40 were medical students. The students had varying experience of simulation and debriefing sessions, from no previous participation to participation on a few occasions. Altogether, seven experienced instructors participated - two males and five females - with varying health professional backgrounds. The instructors have training from Dansk Institut for Medicinsk Simulation (DIMS). The debriefing follows three steps, which is described by Steinwachs [27] and can be found in many models of debriefing. The debriefing sessions generally lasted 15–30 min.

Data was analysed collaboratively by the authors, contributing different backgrounds in healthcare work (such as physiotherapy, biomedical laboratory scientist) and in medical education as well as in education. We developed a model for collaborative analysis of video data. A scheme for a layered, purposeful constant comparative analysis as described by Boeije [28], was adopted for our purposes. The analysis scheme comprised three phases of collaborative activities. The sequence and process of the collaborative analysis are detailed in Table 1.

**Table 1:** Three phases of collaborative analysis of video-recorded debriefings

Phase 1–3 of the analysis	Purpose	Analysis activities	Questions	Results
1. Comparison of multiple perspectives within a single video-recording	Developing collectively enriched and shared understanding of the sequence of activities	Merging individual field notes, reaching consensus on interpretations of fragments	How is debriefing of interprofessional collaboration enacted?	Focus and process for phase 2 determined
				Structural features for comparing video recordings formulated (openings, interaction, closings)
			How are different professional knowings made relevant in the debriefing?	
2. Comparison between different video recordings of the same scenario	Developing a shared understanding of the patterns across the data	Comparing openings, interconnections between human and non-human actors, and closings.	How is the sequence of activities in the debriefing enacted?	Transcripts of selected segments.
				Provisional relational interpretations of the patterns of debriefing as a focus for phase 3 (structure /lack of structure)
			How are socio-material arrangements related to sayings and doings in the debriefing?	
3. Comparison between video recordings of different scenarios	Enlarging and enriching the basis for interpretation.	Refining provisional interpretations against wider data	How is debriefing of interprofessional collaboration enacted across scenarios?	Relational patterns across all data, debriefing as *algorithm* or laissez faire
	Identifying variation			

## Results

The following section shows how simulation practice emerges amongst different cultural-discursive, material-economic and socio-political arrangements into two distinctive patterns of debriefing, labelled as *algorithm or* as laissez-faire. The analysis shows how the emerging debriefing practices are relational to material objects, such as the protocols and arrangements of the debriefing room, but also to ideas of collegiality. Data and analytical descriptors are presented in Tables 2 and 3.

**Table 2:** Keywords and data for debriefing as *algorithm*

Keywords	Field note/quote
- Pre-structured	Field note 1
	The students, dressed in white clinical clothes, drop in after the simulation, sit down around the table and engage in small talk with each other. The instructor enters, dressed in everyday clothes, and all the students turn their attention towards him. The instructor starts: “So we are going to debrief now. We think it is a good idea that you do not discuss the scenario with each other before hand because we believe that we should do it together here in the debriefing. We use a certain model for debriefing. Sometimes when you have done or experienced something you have a tendency to be self-critical and talk about what you could have done differently. We believe that it’s not the best way to analyse this./…/ we usually say that we have three steps. First we talk about what happened, completely factual. Because it is not certain that we all saw what happened [during the scenario] and then we analyse. We do that by first talking about what we did well. We do that so we become aware what it is that I or we are good at, so we can continue doing that and then we can continue to maybe talk about what we could have done differently. And lastly, we also discuss how we can use this scenario, and we will also look a little at a video sequence. But before we get going I want to start by asking how you are feeling at the moment? Johan?” Johan, a nursing student, clears his throat and answers: “Confused… “(Site 2)
- Systematic procedure	
- Protocol steered	
- Instructor centred	
- Close inquiry approach	
- Reinforce good professional performance	
- Strengthening team performance	
	Field note 2
	The group has just summarised the scenario and the instructor says: “OK, now let’s start by talking about what you did well. If we begin with you, who were in the room first, I’m thinking about Thomas and Johanna.” Thomas, a nursing student, starts and says: “Well I think that it would be easier to start at the other end. I came in and well…” The instructor interrupts and asks “At the other end? What do you mean by that?”. Thomas answers “Well, there are things that I could have done differently from the start, but you do not want us to start there”. The instructor nods and says: “No I think that you should raise things that you think were good”. Thomas looks down at the table, sighs and after a while he says: “No I cannot think of anything right now since I feel that there was a lot that I could have done differently”. One of the other nursing students raises her voice and says: “But I think that you gave feedback well and/…/ and the phone call you made was good, if you compared it with the call that I did before.” (Site 2)
	Field note 3
	The students and the instructor have been discussing what the team did well for a while and then the instructor says: “I thought that we would look at a short film sequence now when we have it [refers to the video equipment in the room] all set up. I want you to look at, well, we will show something that we think that you did well, as you know. I want you to look at the structure here, that you think works well.” [a film sequence of 1.41 min is shown] The instructor says: “So what do you say? Did you think of something good that you saw there?” [there is a giggle in the room, then quiet] One of the acting nurse students “Well as we said before, it was very good that Niklas [medical student] made summaries, like we have done this, this and this, and now we are here so that everybody knows.” The instructor nods “Yes it was confident, clear and structured A, B, C, D and E. Did you think about it during the scenario?” One of the nurse students answers: “Yes a little, it felt like we knew what we were doing and maybe because of that it felt very relaxed.” (Site 2)

**Table 3:** Keywords and data for debriefing as laisséz-faire

Keywords	Field note/quote
- Collegial conversation	Field note 4
	The students, dressed in white clinical clothes, burst into the room after the simulation and try to grab a chair. You can hear a voice outside the room saying: “We will need more chairs in here.” The students talk to each other and then the instructor, dressed in green clinical clothes, comes in and while she is trying to find a chair she says: “OK, good folks. So, let’s have free comments from all of you, actors as well as those who observed!” And then she leans back in her chair. One of the medical student starts immediately and says: “Well I still haven’t phoned the doctor on call. I, it is hard to remember to phone the doctor on call. I just, oh yes the doctor on call.” And she and the other students as well as the instructor laugh a bit and the instructor says: “Well absolutely, it could have been a good idea but it went well anyway. Somebody else? “(Site 1)
- Without structure and clear aim	
- Spontaneously	
- Open inquiry approach	
- Ad hoc reflection	
- Reinforce good professional performance	Field note 5
- Focus on future professional practice	Everybody has just sat down around the table and one of the acting nurse students says “When you are standing and working on setting up an IV drip or things like that, then you do not follow what happens up there [refer to the upper part of the patients body] but you keep an overall awareness about what is happening. It felt like we had a good leader keeping control.” Another of the acting nurse students continues: “It was very good that you went through A, B, C all over again and again since the patient’s condition could have deteriorated and then it would be easy to miss if you just stopped. It was also loud and clear [refer to the medical students voice]”. Another nursing student continues: “I, as an observer, thought that you had good team work, that you were calm and that you talked to each other. I didn’t experience that some of you were standing [she makes her body rigid, folds her arms across her chest and just looks around], I think thateverybody was working together and it was clear.” The instructor: “Systematic, yes!”. One of the observing medical students turns to the acting medical students and says: “I thought that you were magnificent but I have some criticisms because there are improvements that you can make. Some things that I can go through quickly. You do not look up when you give an order. You say: start an IV line. I think that it would be good if you [she exemplifies by point to some persons with her hand] so they knew”. The instructor interrupts and says: “Can I stay there? What do those of you who were working in there think about this comment? How was it?” One of the acting nurse student: “Well it was loud and clear but I get what you mean, that somebody really gets an order to do something.” The instructor: “Who is somebody? You do not have somebody written on your name tag so use the names so it is clear.” (Site 1)
	Quote 1
	Instructor 1 argues: “You can do things in different ways [relates back to the scenario where one of the medical students could have delegated the task of calling the doctor on call] and this is the purpose of this day, that you can think I can do this or I can do that. Then you have thought about it once before you are placed in a real situation in a couple of weeks.” (Site 1)

### Debriefing as algorithm

Debriefing as *algorithm*, (Table 2), can be described culturally-discursively as a procedure following a certain protocol. The protocol acts on the practice, producing a pattern of interactions between the participants. These patterns are predominantly pre-defined and descriptive in character. The steps are 1) a brief, uncommented sharing of immediate emotions, 2) description of what happened during the scenario, 3) description of what the team did well, 4) description of what could have been done differently, and 5) implications (field note 1, Table 2). The steps of the protocol are related to how the students’ doings, sayings and relatings during the simulation are raised as topics for discussion in the debriefing session as an algorithm to be followed. The material set-up of the debriefing room acts on how the students position themselves in the debriefing session. The location of the debriefing is similar to that of the briefing session. The students take the same seats around the table as in the briefing, and the instructor similarly takes her/his allocated chair (Table 2, field note 1). The protocol in use emerges through the instructor’s closed inquiry approach, which adheres closely to the pre-defined steps, setting an agenda for the session for what is legitimate to bring up for discussion and reflection. The set-up of the room (such as fixed positioning of chairs, tables, and participants, as well as the use of video clips demonstrating ideal performances of the participants) reinforces the pattern of the algorithm to be followed.

The debriefing practice as *algorithm* displays similarities to models of psychological debriefing as a method to deal with a traumatic experience and crisis. Psychological debriefing follows a specific structure and format, aimed at reducing stress [e.g. 10]. Despite a strong structuring of the turn-taking, the algorithm seems to disturb students’ reflections on their experiences (Table 2, field note 2). A common pattern is that the students are preoccupied by their performance in their professional task and role. The explicit rules of the algorithm, however, constrain what issues are brought back to learners as topics for discussion. During the debriefing only video clips of successful sequences of the simulation are shown. The successful sequences of sayings and doings are related to specific learning objectives and are expressed verbally as empowering examples of good professional practice in the situation (Table 2, field note 2–3). Interestingly, interprofessional collaboration is seldom raised as a topic for discussion. On such occasions, the positive aspects of students’ doings and sayings are emphasised, and negative aspects are downplayed. An illustration of this is when the instructor selects a video clip to exemplify a sequence of good team performance (Table 2, field note 3).

### Debriefing as laissez-faire

*Debriefing as *laissez-faire (Table 3): this procedure can be described culturally-discursively as a collegial conversation without an explicated pre-defined structure or expressed aims. The instructor’s sayings and doings have the characteristics of a chairperson - initiating the conversation and facilitating turn-taking. The instructor’s sayings act on the debriefing practice through an open inquiry approach (Table 3, field note 4), making the reflection participant-driven and loosely structured, based on the sequence of activities and interactions during the simulation. There are no protocols or explicitly communicated learning objectives guiding the debriefing. Students’ experiences of the simulation are re-actualized spontaneously. When students raise issues of importance for them, they construct their own narratives, bringing back experiences from the simulation as topics for reflection in the debriefing. The absence of structured turn-taking acts on the practice in the sense that some student sayings become foregrounded and voiced, while others are back-grounded or even silenced (Table 3, field note 4–5).

The sociomaterial set-up of the debriefing room, a different location than the room used for the briefing session, with swivel chairs around four small round tables and a video screen on one side of the room, prefigures a loosely structured and collegial debriefing practice. Students’ and instructors’ positionings are arbitrary, and the video equipment is never used in the debriefing. The topics raised in the discussion by the students, whether they are positive or negative, are emphasised by the instructors as being valuable experiences, empowering students in professional practice (Table 3, quote 1). The topic of interprofessional collaboration is brought up for reflection only if any of the students raise the issue, or if the instructor raises the issue of communication and leadership. The complex logistics of the simulation involve a large number of students rotating between different stations. The logistics act as a constraint on the debriefing, producing a need for the debriefing session to be completed within approximately 15 min. The time constraint is shown in the instructor’s sayings, as repeated comments on the need to proceed or stop the debriefing.

## Discussion

The literature on simulation and debriefing advocates a multitude of models in the search for how to design the best form of debriefing for learning [29]. A general feature of the models is that they prescribe a defined sequence of phases that the debriefing should follow [6, 27]. Firstly, the participants’ actions during the simulation should be described in the conceptual phase. Secondly, their experience and performance should be analysed, and thirdly, the discussion should consider how the learning could be applied. Dieckmann et al. [11] suggested that instructors might take on personal styles of debriefing that impact on the dynamics and interactions. A sociomaterial perspective, as used in this study, suggests that the patterns of interaction in the debriefing go beyond the personal preferences of the instructor. Debriefing as *algorithm* shows that the prescribed protocol for debriefing emerges in practice as a set of descriptive and normative rules, acting on practice through the interrelated doings, sayings and relatings of the instructors and participants [25].

The different patterns of debriefing, as *algorithm* or as laissez-faire show how debriefing practices vary. The theoretical perspective applied makes it possible to discern how both intentional action and participants’ knowledge of practice shape debriefing. The findings also show how the debriefing shapes and at the same time is shaped by existing circumstances and conditions external to specific debriefing, such as the health care practice. These conditions comprise interrelated cultural-discursive, material-economic and socio-political arrangements that make up practice architectures [25]. These relationships provide meaning and substance to the practice, making certain actions such as interprofessional collaboration more plausible than others. The cultural-discursive arrangement (in terms of values, traditions, and professional language) emerges through the instructors’ and students’ sayings and doings related to the material-economic arrangements (in terms of the set-up of the room and material resources in use). The socio-political arrangements (in terms of relatings between students and instructors) in turn, prefigure professional doings and sayings that become topics for reflection or are neglected in the discussion between students and instructors.

Taken together, the relationships between the arrangements make certain activities more likely to happen [24]. Seen from a pedagogical perspective, this means that sociomaterial arrangements also influence students’ learning. The enactment of debriefing as *algorithm* interrupts the students’ own narratives when bringing experiences during simulation to the debriefing. Instead, the emerging sayings become shaped in a particular way, ruled by defined protocols and values regarding good professional procedures and behaviours. Debriefing as laissez-faire is shaped by a different practice architecture that makes students’ narratives emerge spontaneously. This makes it possible for reflections relevant to students’ experience to emerge in the discussion. Motola et al. [7] emphasise that the feedback in debriefing should be guided by individual learning needs. Debriefing as laissez-faire seems in our study to accommodate for individual learning needs to a greater extent than debriefing as *algorithm.*

However, both patterns of debriefing practice showed that interprofessional learning was rarely brought up for reflection in the discussion, even though the overall aim of the simulation activity was to provide opportunities for students to engage in teamwork and interprofessional collaboration. The findings indicate that both the imposed structure and the lack thereof are cultural-discursive arrangements, and both risk blocking out focus on intended learning outcomes.

The findings of this study support previous research [11–13] in that the instructor’s role in facilitating the group process and involving the individual in the debriefing is found to be important. Kihlgren et al. [8] adopted a previously developed framework for analysing levels of reflection and applied the framework on utterances in 38 debriefing sessions with 10 instructors conducting the debriefing. The findings showed that the participants’ reflections levels were usually low, and that no differences were found in the debriefers’ utterances across occurrences of higher and lower reflection. However, we argue that a focus on individual actors, such as the instructor, is not sufficient for understanding how debriefing practices emerge. Our study suggests that different practice architectures prefigure how the feedback to students is carried out. The feedback emerges in relation to different sociomaterial arrangements that act differently on the practice of the debriefing. Reamer et al. [5], in their review of debriefing research, pointed out a lack of studies comparing debriefing techniques, and studies examining the effects of different debriefing venues and times. Using a sociomaterial perspective as the lens for analysis does not provide causal explanations of the effects of different teaching or venues for debriefing explicitly. However, the focus on practice and findings of our study contribute to an increased understanding of why different debriefing techniques might vary and how the material arrangements of different locations for debriefing are entangled with the emerging practice.

It is also noticeable how the legacy of psychological debriefing of traumatic and stressful experiences structures the debriefing sequence of, and feedback for defusing emotion and stress [10, 14]. When it comes to simulation-based education in undergraduate health care programs, it can be argued that the learners in this context differ from professional practitioners, who participate in simulation as part of their continuing education in the workplace. The health professionals are there to *practice* skills that they have already gained in their previous training. To the health professionals, the acute situation is familiar, but occurs less often, and hence needs to be refreshed under safe conditions. The students, on the other hand are there to *learn* professional skills that they have not yet fully acquired, in a practice situation that might be new to them. This study suggests a difference in purpose and outcomes between a debriefing of a traumatic experience and a debriefing for learning. A focus on defusing emotions might be comforting for the individual, but at the same time, the extent to which professional learning is supported can be questioned. Jeffries [29] suggests that if the aim is to support professional development and learning, it is important to promote a reflective practice in which the students get the opportunity to reflect on their emotions and receive affirmation of their thoughts and actions. The findings of this study indicate that a strong focus on protocols as occurs in debriefing as *algorithm,* and avoidance of the more negative aspects of the enactments of the scenario might constrain opportunities for learning and the necessary uncovering of the reasoning processes behind students’ actions. It is also important to take into account the time constraints imposed by complex logistics of arranging interprofessional simulation-based education for large numbers of students. The time constraint might act negatively on, and jeopardise the potential for learning, both in the structured framing of debriefing as *algorithm* as well as in the loose structure and collegial discussion of debriefing as laissez-faire. Previous research has emphasised the importance of the duration of the debriefing in order to meet the objectives of the simulation and to ensure learning [9, 13]. Seen from a sociomaterial perspective, time can, in this respect, also be viewed as an important actor, related to the processes and outcomes of debriefing practice.

## Conclusion

The findings of this study demonstrate two distinct patterns of enacting debriefing as an activity that is not only dependent on reflection, but entangled with material set-ups, time constraints and social interaction. Neither an imposed structure of the debriefing, nor the lack of structure assured interprofessional collaboration to emerge as a salient topic for reflection, even though that was an explicit learning objective for the simulation. Awareness of how debriefing unfolds and is enacted in practice directs the attention towards sociomaterial dimensions. This kind of broad analysis has revealed multiple elements that inform teamwork and interprofessional practice. The findings thereby contribute to an increased understanding of simulation as an interprofessional learning context and the development of simulation pedagogy.

### Ethics approval and consent to participate

The research project was ethically approved by Linköping University, Sweden (Dnr 2012/439-31). Written informed consent was obtained from all participants.

### Availability of data and materials

Video and other empirical data are stored according to agreements with participants and ethical standards.

### Consent for publication

Not applicable.
